# Antimicrobial activity of cold atmospheric-pressure argon plasma combined with chicory (*Cichorium intybus* L.) extract against *P. aeruginosa* and *E. coli *biofilms

**DOI:** 10.1038/s41598-023-35906-x

**Published:** 2023-06-09

**Authors:** H. Shabani, A. Dezhpour, S. Jafari, M. J. Mehdipour Moghaddam, M. Nilkar

**Affiliations:** 1 Department of Physics, Faculty of Science, University of Guilan, Rasht, 41335-1914 Iran; 2 Department of Biology, Faculty of Science, University of Guilan, Rasht, 41335-1914 Iran; 3 Research Unit Plasma Technology (RUPT), Department of Applied Physics, Faculty of Engineering and Architecture, Ghent University, Sint-Pietersnieuwstraat 41 B4, 9000 Ghent, Belgium

**Keywords:** Biotechnology, Microbiology, Physics

## Abstract

The present study reports a significant combined antibacterial activity of *Cichorium intybus* L. (known as *Chicory*) natural extract with cold atmospheric-pressure argon plasma treatment against multi-drug resistant (MDR) Gram-negative bacteria. To detect reactive species that are generated in the argon plasma, optical emission spectra were recorded. The molecular bands were allocated to the hydroxyl radicals (OH) and neutral nitrogen molecules (N_2_). Moreover, the atomic lines form the emitted spectra were determined to argon atoms (Ar) and the oxygen atoms (O), respectively. The results revealed that *Chicory* extract treatment at a concentration of 0.043 g/ml reduced the metabolic activity of *P. aeruginosa* cells by 42%, while, a reduced metabolic activity of 50.6% was found for *E. coli* biofilms. Moreover, the combination of *Chicory* extract with 3 min Ar-plasma introduced a synergistic effect, so that it exhibited a significantly reduced metabolic activity of *P. aeruginosa* to 84.1%, and *E. coli* ones to 86.7%, respectively. The relationship between cell viability and membrane integrity of *P. aeruginosa* and *E. coli* biofilms treated with *Chicory* extract and argon plasma jet were also analyzed by CLSM. It was found that after the combined treatment, a noticeable membrane disruption was formed. Besides, it was concluded that *E. coli* biofilms showed a higher sensitivity to Ar-plasma than *P. aeruginosa* biofilm at longer plasma exposure times. This study suggests that the anti-biofilm therapy based on a combined effect of *Chicory* extract and cold argon plasma treatment can serve as a considerable green method for treatment of antimicrobial MDR bacteria.

## Introduction

Emerging antibiotic resistance is recognized as one of the most serious public health problems with high mortality rates associated with multi-drug resistant (MDR) bacterial infections^1–3^. A lot of antimicrobial drugs are incapable to control infectious diseases owing to the acquisition of antibiotic resistance. In addition, antibiotic drugs cause numerous biochemical changes and side effects after entering the human body^3, 4^. Due to the increasing threat of MDR pathogens and the ongoing evolution of resistance, there is an urgent requirement for the development of alternative approaches to tackle resistant micro-organisms^4–6^. The new drugs have been produced in the field of infectious diseases by modification of existing drugs to enhance efficacy, minimize toxicity, and reduce resistance^6, 7^. The development of plant extracts-based drugs is a valuable green solution to overcome the deficiencies of conventional drugs^8, 9^. Plant extracts that contain various groups of phytochemicals have great antimicrobial potential against bacteria, fungi and other microorganisms^9^. Medicinal plant extracts exhibit high sensitivity to antibiotic-resistant microorganisms and they are safer than synthetic drugs^10^. Disruption of bacterial membranes through envelope integrity and prevention of succinate dehydrogenase is the key factor in the antibacterial properties of plant extracts^10, 11^.

In recent years, the utilization of antibacterial plant extract combined with the therapeutic use of cold atmospheric-pressure plasma has been represented remarkable alternatives to antibiotics therapies^12–14^. Cold plasma represents many desirable features involving relatively low operational cost, operation at the ambient temperature, tunable output chemistry, high inactivation efficacy, and no toxic residues^15, 16^. The influence of cold plasma depends on the action of the generation of chemically reactive species including reactive oxygen and nitrogen species like OH, O, O_3_, N_2_, charged particles, and UV radiation^17–19^. The interaction between the produced active species in plasma with surrounding moisture and H_2_O molecule leads to the production of H_2_O_2_, NO_2_^−^, and NO_3_^−^ in liquid phase of the targeted surface^20^. Some of them possess high reduction potential that can eventually lead to the cell death in micro-organisms. In anti-biofilm therapy based on a combined effect of plant extract and cold plasma, the antibacterial agents produced in plasma can give rise to synergistic effects^21, 22^. Since the plasma species are highly reactive and can potentially exert considerable oxidative stress they can damage to cells through lipid peroxidation and enzyme inactivation^23^. Lipid peroxidation can be harmful to living cells by changing their membrane properties through perturbing its integrity and permeability^24^. In fact, it could modify the functionality of the antibacterial compounds of the extract combined with cold plasma resulting in to increase the phenolic content, especially against drug resistance mechanisms^25, 26^.

Among Gram-negative bacteria, *Pseudomonas aeruginosa*, as one of the most serious causes of healthcare-related infections, plays an important role in diseases such as urinary tract infections, endocarditis, burns, and wound infections^27^. On the other hand, *Escherichia coli* is the most common Gram-negative bacterial pathogen among resistant bacteria and causes a diverse range of diseases affecting all age groups^28^. The resistance of these two biofilms to numerous antibiotics is limiting therapeutic options. For Gram negative bacteria like *P. aeruginosa* and *E. coli*, which have a thin cytoplasmic membrane and cellular wall, once the damaged envelope has lost its osmotic capacity due to the cold plasma treatment, intracellular content leaches, which induces irreversible damages and cell death^23, 24, 29^. In the past decade, some research groups have found promising results on the synergistic effect of cold plasma treatment on the plant extracts^12–14, 21, 22, 25, 30–32^. In this regard, Shu et al.^22^ investigated the antibacterial and cytotoxic activities of plasma-modified polyethylene terephthalate nonwoven dressing with aqueous extract of Rhizome *Atractylodes macrocephala*. The results revealed that the plasma-treated extract increased the broad-spectrum antibacterial effects of PET dressings containing RAM extract against *E. coli* and *S. aureus* bacteria which was no less than ones of trade antibacterial dressings. Vajpayee et al.^25^ showed the antimicrobial activity of air plasma-treated banana fabric coated with natural leaf extracts. The authors found that air plasma treatment followed by the absorption of green tea (*Camellia sinensis*) and tulsi (*Ocimum sanctum*) leaf extracts can assist as a valuable method for developing natural antimicrobial textiles in medical and healthcare sectors. Matan et al.^21^ investigated the combined antibacterial activity of green tea extract with atmospheric radio-frequency plasma against pathogens (like *E. coli* and *S. typhimurium*) on dragon fruit. The research indicated that higher values of total phenolic content and crude protein were observed in the fresh-cut dragon fruit with green tea after the plasma treatment.

In this study, for the first time, we aim to investigate a combined effect of *Cichorium intybus* L. (known as *Chicory*) extract with cold atmospheric-pressure argon plasma treatment against MDR *P. aeruginosa* and *E. coli* bacteria. *Chicory* extract has abundant variety of chemical compounds that warrant it a broad range of uses, rendering action against bacteria, viruses and fungi^33^. This herbal extract has revealed promising antibacterial traits against both kinds of bacteria^34^. In addition, *Chicory* extract has valuable therapeutic properties such as antidiabetic, antioxidant, and anti-inflammatory^35^. Indeed, phenolic and polyphenolic compounds found in *Chicory* extract make up the essential function used to fighting MDR bacteria^34, 35^. The mechanisms of influence of phenolic compounds against bacteria involve destruction of cytoplasmatic membrane and inhibition of DNA as well as adenosine triphosphate-related enzymes^36^. As we will show, synergistic and additive interactions are a consequence of a combined effect of *Chicory* extract and active species from argon plasma against MDR *P. aeruginosa* and *E. coli*.

## Materials and methods

### Preparation for *P. aeruginosa* and *E. coli*

A detailed description of preparing the bacterial strains and the formation of *P. aeruginosa* and *E. coli* biofilms is mentioned in our previous studies^2, 6, 37^. In short, two bacterial strains including *P. aeruginosa* ATCC 27853 and *E. coli* ATCC 8739 were used. A colony of each bacterial strain was cultured in a nutrient agar medium at 37 °C for 24 h. Bacterial concentration was set to 0.5 McFarland. Dilution was done to reach the desired concentration (1.5 × 10^6^ CFU/mL). Bacteria were added to the wells containing the Mueller Hinton Broth culture medium and placed in an incubator at 37 °C for 48 h to form the biofilms. After 2 days, the biofilm samples were washed three times with sterilized distilled water to remove excess liquid and leave only the biofilm layer. Finally, the samples were treated with *Chicory* alcoholic extract and atmospheric pressure cold argon plasma for 10 s, 90 s, and 180 s. After treating the biofilm samples, plates were stained using crystal violet solution so that the absorption coefficient can be read by the reading device. The amount of staining procedure is proportional to the number of adherent cells in the sample. Therefore, the wells were washed using sterilized distilled water and stained with 100 μL of 0.1% crystal violet solution for 15 min at room temperature. The plates were washed using sterilized distilled water and then left to dry. Finally, 100 μL of 30% acetic acid was added to each well to solubilize the dye and the OD_630_ was recorded using a microplate reader (Bio-Rad, USA).

### Plant material and preparation of herb extract

To prepare the alcoholic extract, at first, 5 g of *Chicory* plant powder should be dissolved in 50 ml of 80% ethanol and then placed in a shaker at 25 °C with 100 rpm for 3 days. Afterward, it should be centrifuged at 8000 rpm for 15 min until the powder is completely separated from the liquid. Then, the supernatant liquid is separated from the powder and dried by a freeze dryer. Finally, 2.5 ml DMSO, 5%, is added to the obtained substance from *Chicory* and passed through a 0.45 µm filter. The extract was obtained with a concentration of 0.043 g/ml. An aqueous solution containing 0.1% crystal violet was also prepared and added to the treated biofilms (with *Chicory* extract and/or argon plasma treatment) to monitor the biofilm formation capacity. In addition, a combination of *Chicory* extract and argon plasma treatments were used in such a way that first the extract and then the plasma treatments were applied on the surface of the samples.

### Plasma source

In this study, we employed a cold atmospheric-pressure plasma jet, and a detailed description of that is illustrated in Ref.^2^. In this configuration, the discharge was triggered by an AC voltage (25 kHz and 8 kV, peak value). Argon was the working gas and the typical flow rate was 2 slm. The ionized gas flowed away from the main discharge area and enters the air and finally forms the plenty of reactive species (i.e., RONS). The plasma plume temperature was measured by a fiber thermometer (FISO FOT-L-SD) with the distance 10 mm from the tip of the fiber. The plasma temperature was approximately 43 °C. In addition, the length and the diameter of the plasma plume were 10 mm and 3 mm, respectively. The distance between the biofilms and plasma jet nozzle was 10 mm under the conditions of our laboratory at 57% relative humidity and temperature 25 °C.

### Plasma spectroscopy

Plasma spectroscopy was used to investigate the cold plasma characteristics. Ocean Optics HR 2000 spectrometer was employed to collect optical emission and thereafter to determine the plasma constituent species. The optical spectra were recorded for emission from the plasma jet in the wavelength range from 200 to 1000 nm with an optical resolution of 0.5 nm. The light emitted by the argon plasma was focused by means of optical fiber into entrance slit of 0.75 m monochromator, equipped with a grating of 2400 grooves per millimeter and slit width of 100 μm. The optical fiber probe was installed 10 mm away from the plasma jet nozzle.

### Hydrogen peroxide, nitrite and nitrate contents in the plasma activated water

To measure the concentrations of hydrogen peroxide (H_2_O_2_), nitrite (NO_2_^−^), and nitrate (NO_3_^−^) in DI water treated by argon plasma jet, spectrophotometric methods (Shimadzu UV–1800 Japan) were performed. At first, 25 mL of DI water in glass was treated with Ar plasma jet with treatment times of 10 s, 90 s and 180 s. The distance between the surface of water and plasma jet nozzle was 10 mm. Then, the H_2_O_2_ concentration was determined by its reaction with Titanyl ions of Titanyl sulfate (TiOSO_4_) resulting into a yellow-colored product with maximum absorbance peak at 407 nm^38^. The NO_2_^−^ and NO_3_^−^ concentrations were determined by the commercial kit using Griess reagents (Cayman Chemicals, MI, USA) forming a pink-colored product with maximum absorbance peak at 540 nm^38, 39^.

### LIVE/DEAD bacterial viability assay and CLSM

To evaluate the live/dead states of *P. aeruginosa* and *E. coli* biofilms, the BacLight™ Live/Dead bacterial viability kit was used in this study. Bacterial biofilm was prepared on microscope cover glasses and the glasses were covered with 240 µl of SYTO®9 (green fluorescence, labeling bacteria with intact membrane structure), and incubated aerobically at 37 °C. After incubation, the biofilms was rinsed gently using phosphate buffered saline to remove planktonic and loosely attached bacteria. For the confocal laser scanning microscopy (CLSM) analysis, bacterial biofilm was prepared on glass slides and then was exposed to *Chicory* extract and argon plasma in different exposure times. In continue, a volume of 100 μL the DNA-binding dye, SYTO®9, was added to the treated biofilms. The slides were incubated in darkness for 20 min at room temperature. Finally, the slides were covered with cover glass. Live/dead cells were detected by CLSM (Leica TCS SP8 X, Wetzlar, Germany) using appropriate filters with excitation/emission wavelengths at 485/530 nm for SYTO®9. The green fluorescence indicates that the bacterial membrane was intact.

### Scanning electron microscopy (FE-SEM) and atomic force microscopy (AFM)

The morphological structure of the biofilm samples (both untreated and cold plasma-treated extract) was analyzed using scanning electron microscopy (FEG-SEM, MIRA3 TESCAN) operating at 15 kV. In addition, the surface morphology was examined using AFM (Model: Agilent 5100) for the scan area of 5 μm × 5 μm using a silicon probe under tapping mode, and the analysis was also done using Nanoscope Analysis software.

## Results and discussions

**Figure 1:**
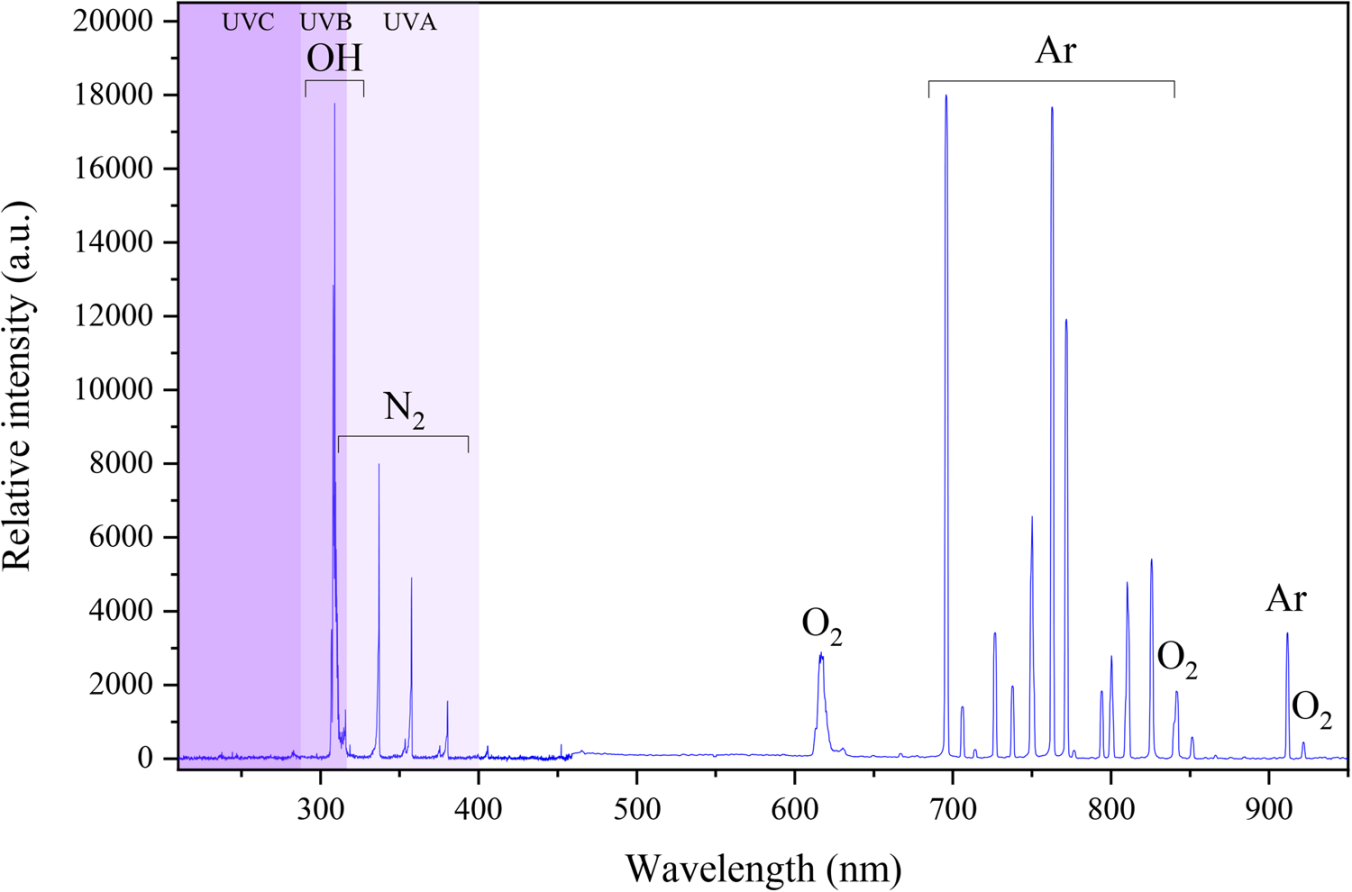
Emission spectra from the Ar-plasma jet with the applied voltage of 8 kV and frequency 25 kHz.

To detect reactive species that are generated in the discharge zone, optical spectra were recorded for emission from the plasma jet in the wavelength range from 200 to 1000 nm. Figure 1 shows the emission spectrum observed in the cold argon plasma jet. The molecular bands were allocated to hydroxyl radicals (OH), and neutral nitrogen molecules (N_2_). The OH radical is detected between 305 and 310 nm regions. Bands of N_2_ are apparent in the spectral range of 310–385 nm. The atomic lines form the emitted spectra are determined to argon atoms (lines between 670–810 nm and 918 nm) and oxygen atoms (lines at 615 nm, 815–850 nm, and 928 nm) as a consequence of the ambient O_2_ and H_2_O dissociation^40–42^.

**Figure 2:**
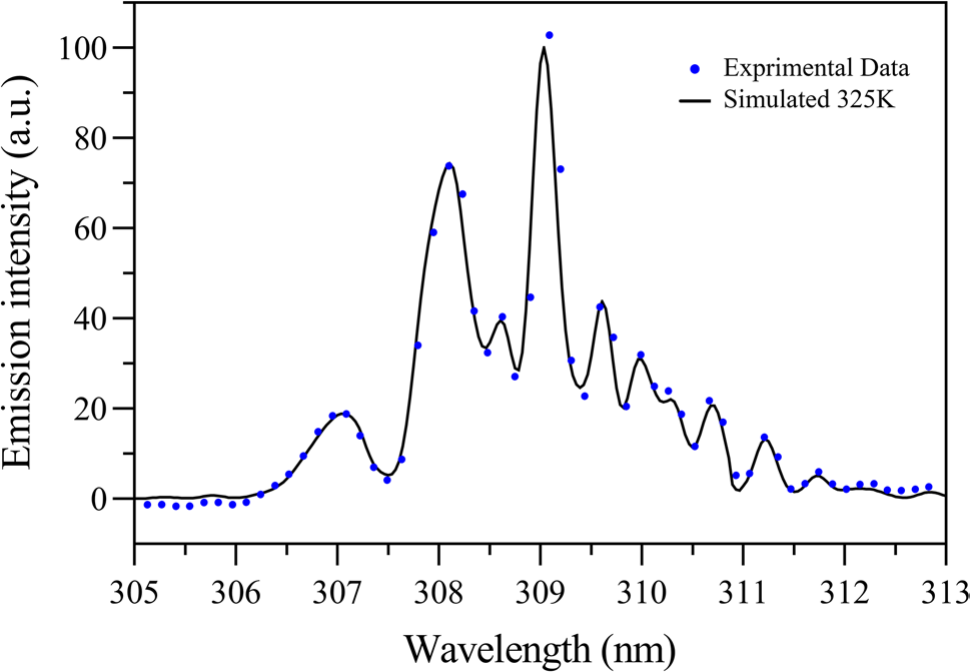
Measured and simulated optical emission spectra with the use of LIFBASE software around the OH for the atmospheric pressure argon plasma jet.

To measure the gas temperature optical emission spectroscopy is an often used non-invasive method. The rotational spectrum of molecules such as OH is widely used for temperature determination^43^. The gas temperature of the cold plasma jet was estimated using the emission spectra of OH (A^2^Σ → X^2^Π) radicals. In this study, the rotational temperatures of OH radicals are considered to be the gas temperature of Ar-plasma. LIFBASE simulation software program was employed to simulate the OH band at a specified temperature. The temperature was estimated by comparing the simulated spectrum with the experimental data. The best fit between the experiment and the simulation reveals the rotational temperature of the experiment^43, 44^. Therefore, assuming that the rotational temperature is equal to the gas temperature, the gas temperature can be properly measured with this method. Figure 2 shows measured and simulated optical emission spectra with the use of LIFBASE around the OH for the atmospheric pressure argon plasma jet. As seen in this figure, the experimental data and the simulation data correspond with each other very well at 325 K with an accuracy of ± 7 K. Moreover, as an alternative temperature measurement, the gas temperature obtained under the same condition by using a fiber thermometer is 316 K. The difference between them is acceptable in our case. The OH rotational temperature is very close to the gas temperature.

**Figure 3:**
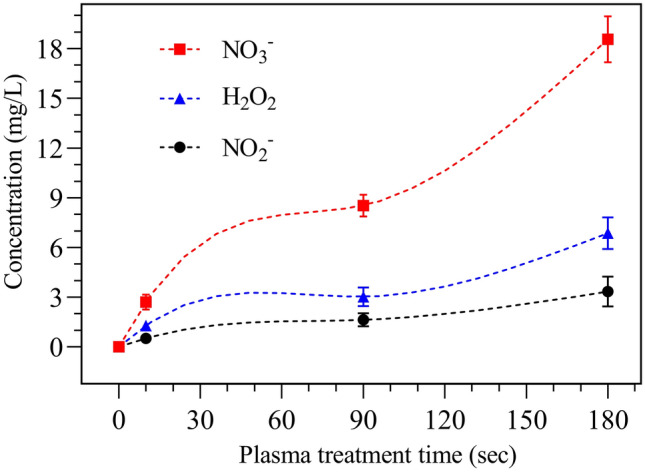
Relationship between the argon plasma exposure times and concentrations of H_2_O_2_, NO_2_^−^ and NO_3_^−^ generated in DI water.

Plasma treatment of liquids leads to the generation of different chemical species, such as H_2_O_2_, NO_2_^−^, and NO_3_^−^^45^. H_2_O_2_ is one of the most common long lived reactive species in plasma activated water (PAW), with its multifunctional activities in cell redox signaling pathways, cell oxidative stress and pathogen inactivation^46^. The formation of H_2_O_2_ is mainly from two pathways: (i) direct transfer of H_2_O_2_ from gaseous plasmas, and (ii) direct recombination of aqueous OH radicals dissolved from the gas phase and/or reactions among other reactive species in the liquid phase^45, 46^. In this study, to determine the concentrations of H_2_O_2_, NO_2_^−^ and NO_3_^−^ in DI water generated by argon plasma jet, the spectrophotometric method was performed. The results are given in Fig. 3. As seen in this figure, a longer plasma exposure time led to higher concentrations of H_2_O_2_, NO_2_^−^ and NO_3_^−^ in DI water. Moreover, for every plasma treatment time, the concentration of NO_3_^−^ was higher than that of H_2_O_2_ and NO_2_^−^. For the plasma treatment time of 180 s, the concentrations of NO_2_^−^, H_2_O_2_ and NO_3_^−^ were between 3.5 mg/L, 6.7 mg/L and 18.2 mg/L, respectively. While studies show different plasma species to be responsible for killing of bacteria, it has been suggested that bacterial killing occurs via three different mechanisms^46–48^: (i) direct permeabilisation of the cell membrane or wall, leading to leakage of cellular components, containing potassium, nucleic acid and proteins; (ii) critical damage of intracellular proteins from oxidative or nitrosative species; and (iii) direct chemical DNA damage. In many cases, plasma-generated reactive species and specially H_2_O_2_ were found to be the causative agent of cell death. In this regard, H_2_O_2_ is a well-known antibacterial agent that damages iron–sulphur and mononuclear iron enzymes in bacterial cells^48–50^.

**Figure 4:**
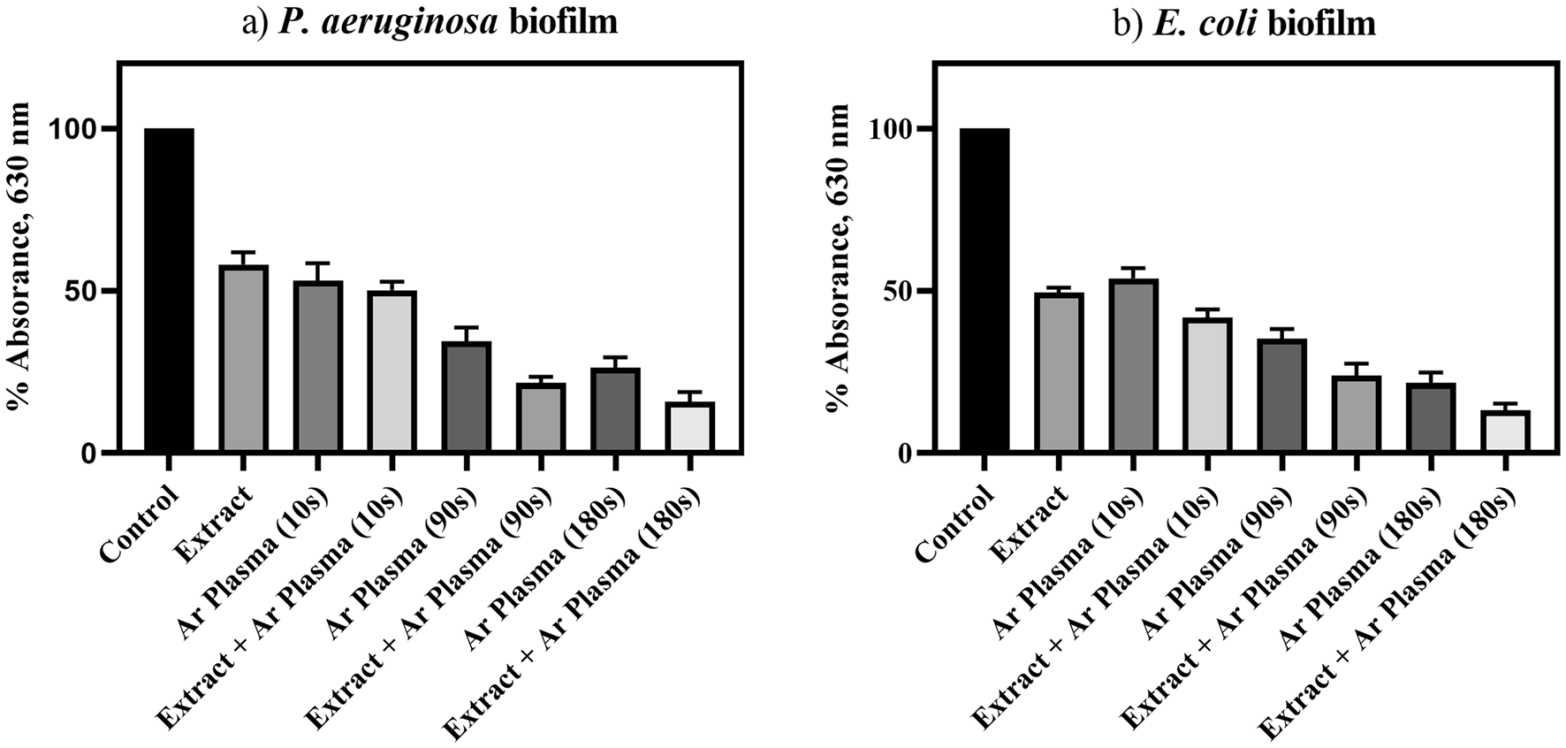
Crystal violet assay to determine the biofilm formation capacity in the cases treated with *Chicory* extract alone or combined with Ar-plasma at different exposure times for; (**a**) *P. aeruginosa* and (**b**) *E. coli*.

Crystal violet (CV) staining was performed to examine the biofilm formation capacity. The effects of survival and metabolic activity of *P. aeruginosa* and *E. coli* biofilms, 48 h after treatment are demonstrated in Fig. 4. According to this figure, *Chicory* extract treatment at a concentration of 0.043 g/ml reduced moderately the metabolic activity of *P. aeruginosa* cells by 42% compared to the untreated control. Similarly, reduced metabolic activity of 50.6% was found for *E. coli* biofilms. The phenolic and polyphenolic compounds originated from *Chicory* extract form the basis of the fight against these biofilms. In addition, using Ar-plasma treatment at three exposure times of 10 s, 90 s, and 180 s remarkably diminished the metabolic activity of *P. aeruginosa* to 46.9%, 65.7%, and 73.8%, respectively, and also *E. coli* ones to 46.2%, 64.9%, 76.1%, respectively. But, a significant decrease in cell metabolic activity was observed after the combination of the *Chicory* extract and argon plasma treatment. CV absorbance values exhibited the synergism of *Chicory* extract with Ar-plasma in the inactivation of the biofilms with increasing plasma exposure time, so that for 10 s, 90 s, and 180 s of Ar-plasma exposure, respectively, 49.8%, 78.5% and 84.1% (for *P. aeruginosa*), and 58.3%, 76.3%, 86.7% (for *E. coli*) were effective in reducing their metabolic activities.

**Figure 5:**
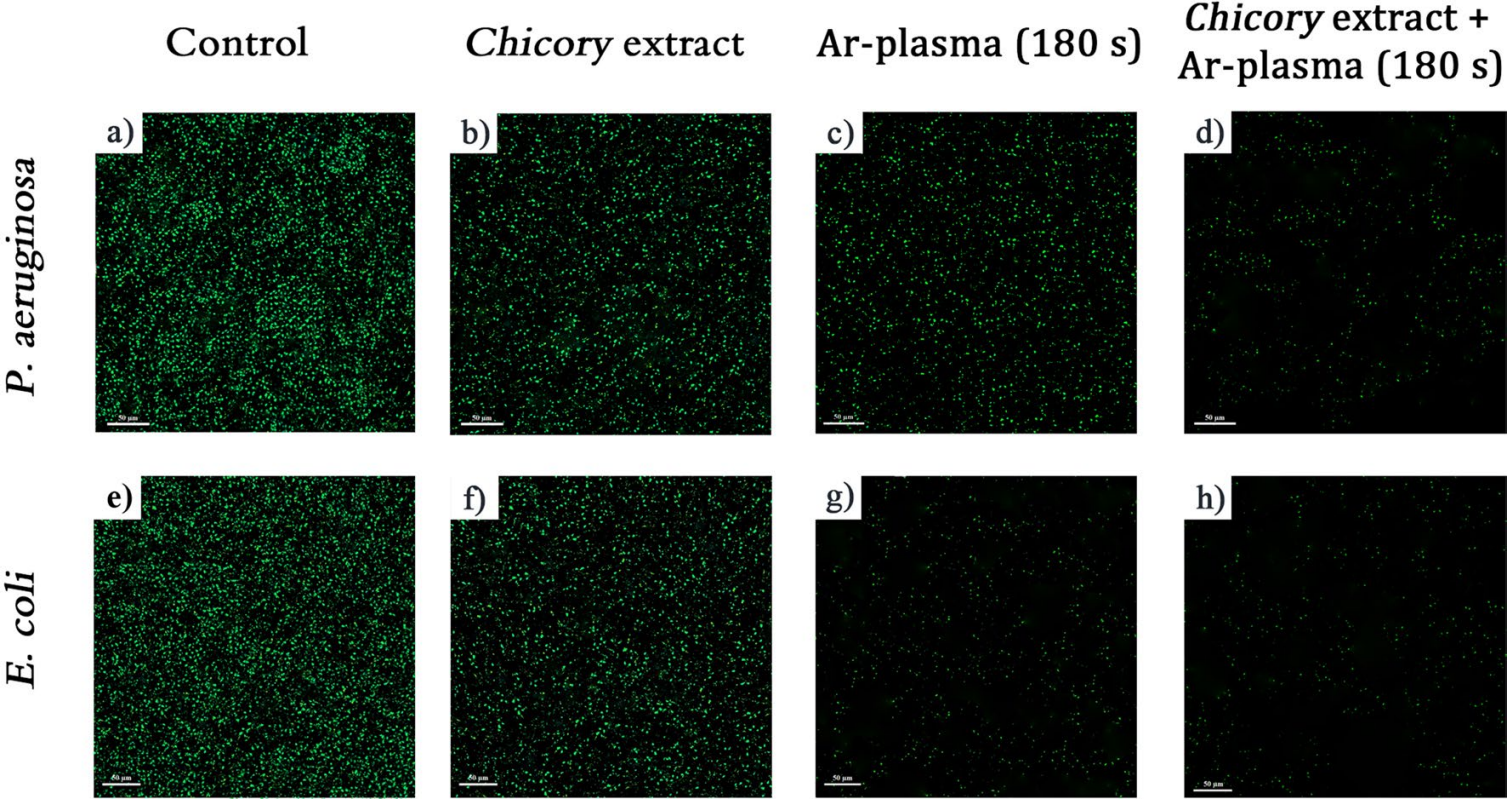
CLSM images of *P. aeruginosa* and *E. coli* biofilms treated with *Chicory* extract alone or combined with Ar-plasma: (**a** and **e**) Control (**b** and **f**) *Chicory* extract (**c** and **g**) 180 s Ar-plasma, and (d and h) *Chicory* extract combined with 180 s Ar-plasma.

The relationship between cell viability and membrane integrity of *P. aeruginosa* and *E. coli* biofilms treated with *Chicory* extract and argon plasma jet were analyzed by CLSM. Figure 5a and e show the CLSM images of the untreated samples and the rest of the images are related to the treated cases. The green-fluorescence stain SYTO®9 can diffuse through intact bacterial membranes. For both bacterial species, the control group with intact bacterial membranes generally showed green florescence cells, except for a few naturally occurring dead bacteria. As is observed in Fig. 5b and f, following the *Chicory* extract treatment, the bacterial cells were lysed and proportion of green fluorescent cells were reduced. Disruption of bacterial membranes and prevention of succinate dehydrogenase through the *Chicory* extract treatment are the key factors in cell lysis. Figure 5c and g are related to the antibacterial effect caused by only argon plasma treatment in 3 min. Although treating the biofilms with Ar-plasma alone reduced bacterial biofilms, some bacterial cells were still attached to the surface indicating the incomplete eradication of bacterial biofilm. It was found that the number of nonviable cells was depended on the time of the Ar-plasma exposure. Besides, it was concluded that *E. coli* biofilms exhibit higher sensitivity to Ar-plasma than *P. aeruginosa* biofilm at longer plasma exposure times. Figure 5d and h show the anti-biofilm effect caused by the combination of the *Chicory* extract and argon plasma treatment in 3 min. Interestingly, the results revealed that the combined treatment of the biofilms with the extract + plasma (3 min) remarkably reduced the frequency of the bright green dots, indicating the major eradication of bacterial biofilms. Synergistic antibacterial effects in the extract + plasma treatment can have been attributed to the existence of both phenolics and RONS diffused by Ar-plasma, which permit intracellular toxins access to their targets by both disrupting bacterial membrane and blocking toxins-removal efflux pumps of specialized strains. Moreover, the extract + plasma treatment may induce bacterial oxidative stress and prompt the production of intracellular reactive oxygen species (ROS) in the biofilms, which possibly contributes to bacteria death. As a result, these synergistic effects could enhance the functionality of the antibacterial compounds, particularly against drug resistance mechanisms.

**Figure 6:**
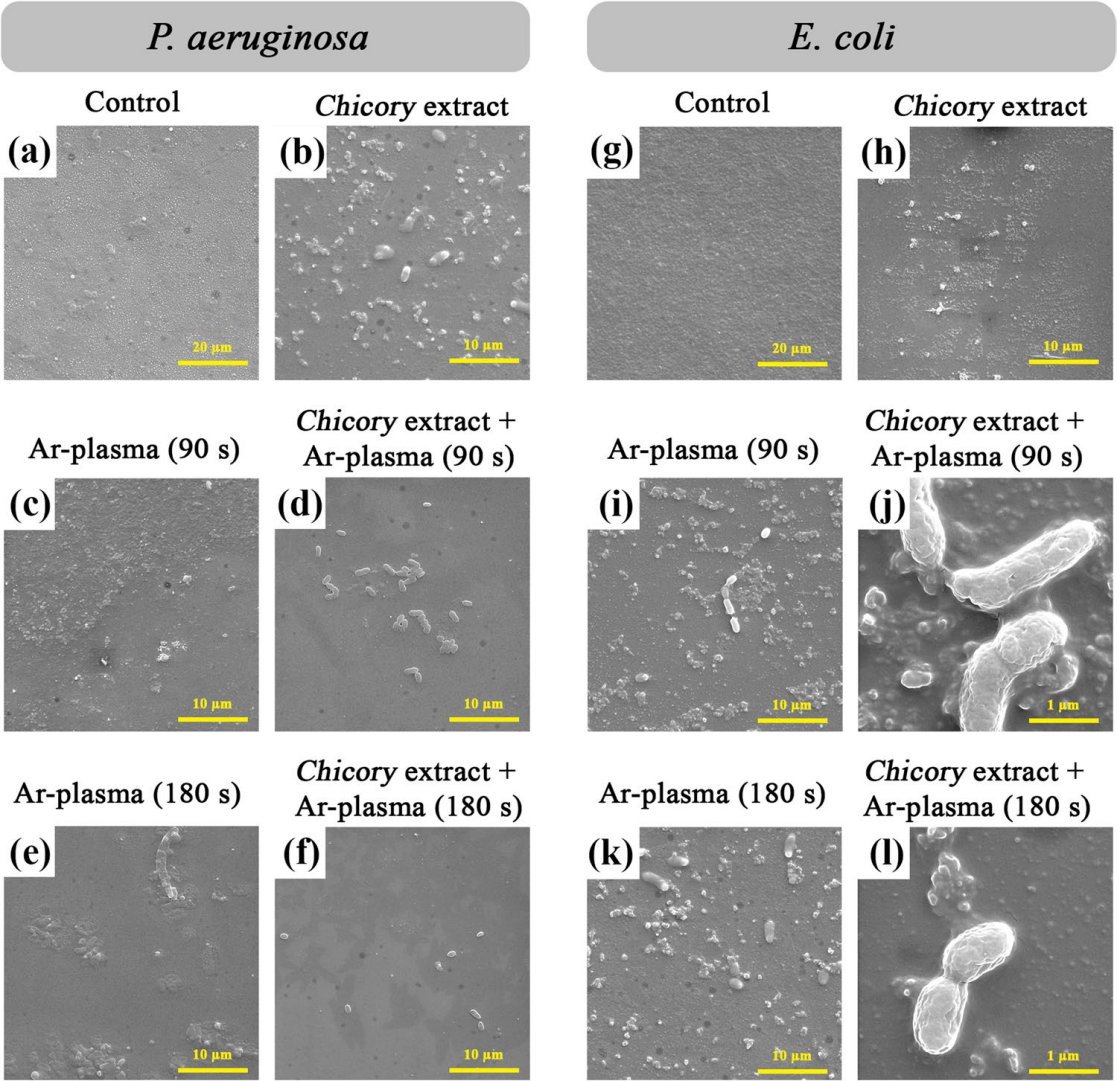
FE-SEM images of *P. aeruginosa* and *E. coli* biofilms treated with *Chicory* extract alone or combined with Ar-plasma at different exposure times: (**a** and **g**) Control, (**b** and **h**) *Chicory* extract, (**c** and **i**) 90 s Ar-plasma, (**d** and **j**) *Chicory* extract combined with 90 s Ar-plasma, (**e** and **k**) 180 s Ar-plasma, and (**f** and **l**) *Chicory* extract combined with 180 s Ar-plasma.

Figure 6 shows the FE-SEM micrographs of *P. aeruginosa* and *E. coli* biofilms treated with *Chicory* extract alone or combined with Ar-plasma at different plasma exposure times. Figure 6a and g are related to the untreated control samples and the rest of the images show the treated cases. As could be observed in Fig. 6b and h, through the *Chicory* extract treatment, the bacterial cells were lysed and cell debris remained on the surface of the plate. Figure 6c and i are related to the anti-biofilm effect caused by just argon plasma treatment in 90 s. Following Ar-plasma treatment, cytoplasmic contents and extracellular polymers of the bacterial cells were gradually released so that the intact viable bacterial cells were hardly observed after the plasma exposure. In fact, after Ar-plasma treatment, the reactive oxygen and nitrogen species (RONS) generated in the plasma plume attack both the cell envelope and intracellular components of *P. aeruginosa* and *E. coli* biofilms. The reactions of RONS with cell components cause disruption of the cell envelope and result in leakage, with some possible damage to intracellular components (e.g., DNA). In addition, as is shown in Fig. 6e and k, the longer treatment time with only Ar-plasma lead to greater bacterial cell lysis and therefore, more efficient biofilm eradication. One can conclude that the plasma radicals and ions bombard the surface of the biofilm, which results in the destruction of the cytoplasmatic membrane and also the etched surface. Figure 6d and j show the anti-biofilm activity caused by the combination of the *Chicory* extract and argon plasma treatment in 90 s. As could be observed in these images, following the extract + plasma treatment for 90 s, intact bacterial cells are hardly observed, suggesting major part rapture of the bacterial cell wall and biofilm eradication. Synergistic and additive interactions are a consequence of a combined effect of active species from the plant extract and argon plasma. Figure 6f and l demonstrate the anti-biofilm effect caused by the combination of the *Chicory* extract and argon plasma treatment in 3 min. As seen in these images, employing longer time exposure of Ar-plasma (3 min) could reduce the significant number of bacteria than using a shorter time of exposure to Ar-plasma. In this test, the combination of the extract with Ar-plasma treatment at 3 min was found to almost completely inhibit all bacteria on the biofilm surface. In the extract + plasma treatment, the phytocompounds disturb the cell wall of the biofilm which leads to intensified permeability of the cytoplasmic membrane and thereby facilitating the influx of the plasma radical species. Another kind of synergistic effect from extract + plasma treatment includes the prohibition of the arrival of environmental carbon sources to bacterial communities by the formation of non-metabolizable compounds for bacteria.

**Figure 7:**
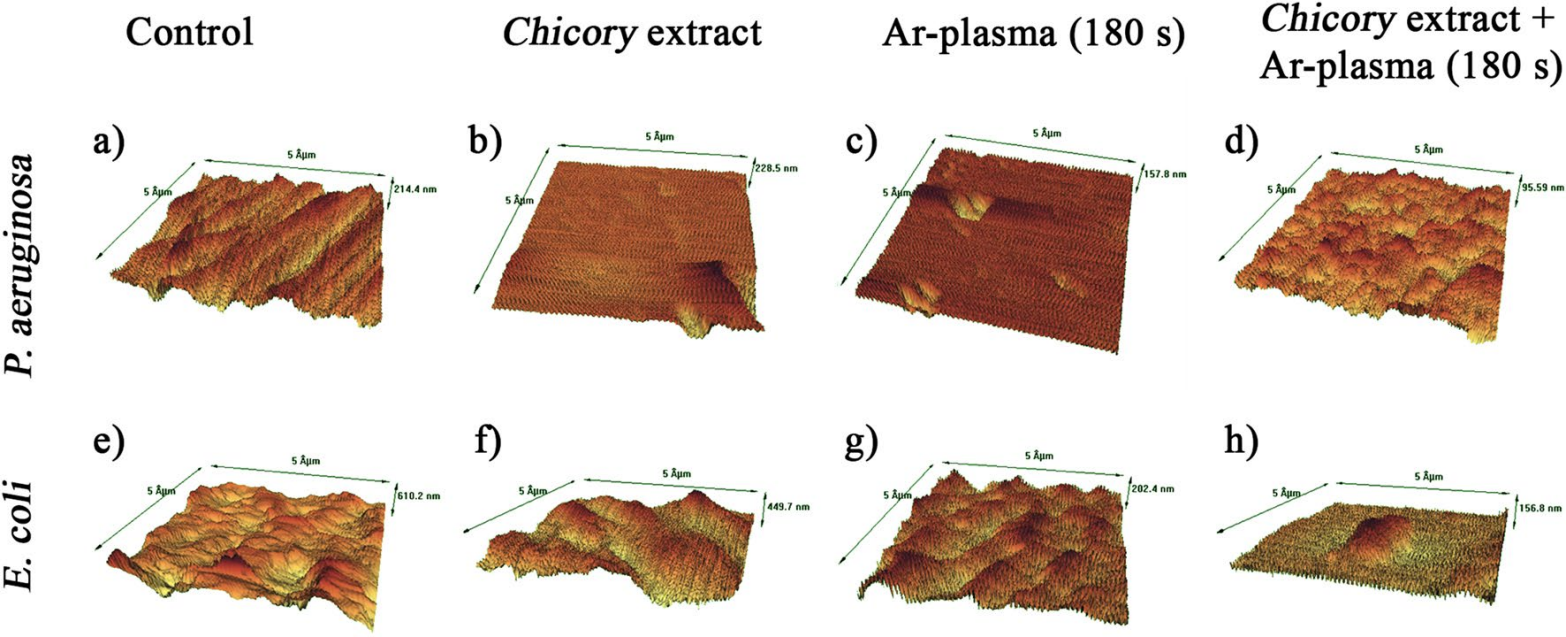
AFM images of *P. aeruginosa* and *E. coli* biofilms treated with *Chicory* extract alone or combined with Ar-plasma: (**a** and **e**) Control, (**b** and **f**) *Chicory* extract, (**c** and **g**) 180 s Ar-plasma, and (**d** and **h**) *Chicory* extract combined with 180 s Ar-plasma.

The AFM technique helps to observe differences in the surface topography of the plant extract or plasma-treated and untreated samples. Figure 7a and e show the control samples and the rest of the images are related to the treated cases. As is observed in Fig. 7b and f, following the *Chicory* extract treatment, the bacterial cells were lysed and cell debris remained on the surface of the plate. Subsequently, *P. aeruginosa* and *E. coli* biofilms show a smoother surface with fewer dips and bumps than the untreated case. Figure 7c and g are related to the antibacterial effect caused by only argon plasma treatment in 3 min. As seen in these images, they represent a relatively etched surface. The etching effect originating from argon plasma exposure could be due to the interaction of ions of plasma with the biofilm along with certain oxidative reactions with activated oxygen atoms. Figure 7d and h show the anti-biofilm effect caused by the combination of the *Chicory* extract and argon plasma treatment in 3 min. Interestingly, after the extract + plasma (3 min) treatment, the images depict significant surface changes involving multiple microcracks on the surface, and it appears that the surface got eroded. These microcracks and the eroded surface were arisen from the longtime direct attack of ions and active species of the cold plasma.

## Conclusions

In summary, the aim of this study was to explore an innovative non-antibiotic approach to combat MDR Gram-negative bacteria based on a combined effect of *Chicory* extract with cold argon plasma. The MDR bacterial infections, like *P. aeruginosa* and *E. coli*, have become a serious worldwide health problem and as a consequence, that issue makes essential the evolution of novel therapeutics. To detect reactive species that are generated in the argon plasma zone, optical emission spectra were recorded. The molecular bands were allocated to hydroxyl radicals (OH), and neutral nitrogen molecules (N_2_). Moreover, the atomic lines from the emitted spectra were determined to be argon atoms (Ar) and oxygen atoms (O), respectively. The gas temperature of the cold plasma jet was estimated using the emission spectra of OH (A^2^Σ → X^2^Π) radicals. In this study, the rotational temperatures of OH radicals were considered to be the gas temperature of Ar-plasma. LIFBASE simulation software program was employed to simulate the OH band at a specified temperature. In addition, to determine the concentrations of H_2_O_2_, NO_2_^−^ and NO_3_^−^ in the plasma activated water (PAW), spectrophotometric measurements were performed. The results indicated that a longer plasma exposure time led to higher concentrations of H_2_O_2_, NO_2_^−^ and NO_3_^−^ in DI water. Moreover, for every plasma treatment time, the concentration of NO_3_^−^ was higher than that of H_2_O_2_ and NO_2_^−^. Further results indicated that *Chicory* extract treatment at a concentration of 0.043 g/ml reduced the metabolic activity of *P. aeruginosa* cells by 42%, while, a reduced metabolic activity of 50.6% was found for *E. coli* biofilms. Moreover, Ar-plasma treatment at 180 s remarkably diminished the metabolic activity of *P. aeruginosa* to 73.8%, and also *E. coli* to 76.1%. Notably, the combination of *Chicory* extract with argon plasma introduced a synergistic effect, so that the extract + plasma (3 min) treatment exhibited a significantly reduced metabolic activity of *P. aeruginosa* to 84.1%, and also *E. coli* ones to 86.7%. Synergistic antibacterial effects in the extract + plasma treatment can be attributed to the existence of both phenolics and RONS diffused by Ar-plasma which permit intracellular toxins access to their targets by both disrupting bacterial membrane and blocking toxins-removal efflux pumps of specialized strains. The relationship between cell viability and membrane integrity of *P. aeruginosa* and *E. coli* biofilms treated with *Chicory* extract and argon plasma jet were also analyzed by CLSM. It was found that after the extract + plasma (3 min) treatment, a significant reduced proportion of green fluorescent cells was formed, indicating remarkable membrane disruption. Besides, it was concluded that *E. coli* biofilms show higher sensitivity to Ar-plasma than *P. aeruginosa* biofilm at longer plasma exposure times. Based on the CLSM and FE-SEM images, it was found that following Ar-plasma treatment (especially for a longer treatment time of plasma exposure), cytoplasmic contents and extracellular polymers of the bacterial cells were lysed so that, the reactions of RONS arising from plasma with cell components led to intracellular oxidation and peroxidation. Moreover, based on AFM images, it was found that after the extract + plasma treatment, significant surface changes involving multiple microcracks on the biofilm surfaces were created. Our results indicated that the microbial inactivation based on a synergistic effect of the *Chicory* extract and cold argon plasma is one of the low-cost and efficacy green strategies having a great potential for control of hazardous biofilms of a broad spectrum of MDR bacteria.

## Data Availability

The data that supports the findings of this study are available within the article.
